# Sleep Deprivation Reveals Altered Brain Perfusion Patterns in Somnambulism

**DOI:** 10.1371/journal.pone.0133474

**Published:** 2015-08-04

**Authors:** Thien Thanh Dang-Vu, Antonio Zadra, Marc-Antoine Labelle, Dominique Petit, Jean-Paul Soucy, Jacques Montplaisir

**Affiliations:** 1 Center for Advanced Research in Sleep Medicine, Hôpital du Sacré-Cœur de Montréal, Montréal, Quebec, Canada; 2 Center for Studies in Behavioral Neurobiology, PERFORM Center & Department of Exercise Science, Concordia University, Montréal, Quebec, Canada; 3 Centre de Recherche de l’Institut Universitaire de Gériatrie de Montréal and Department of Neurosciences, Université de Montréal, Montréal, Quebec, Canada; 4 Department of Psychology, Université de Montréal, Montréal, Quebec, Canada; 5 Montreal Neurological Institute, McGill University, Montréal, Quebec, Canada; 6 Department of Nuclear Medicine, Centre Hospitalier de l’Université de Montréal, Montréal, Québec, Canada; 7 Department of Psychiatry and Canada Research Chair in Sleep Medicine, Université de Montréal, Montréal, Quebec, Canada; Oasi research institute, ITALY

## Abstract

**Background:**

Despite its high prevalence, relatively little is known about the pathophysiology of somnambulism. Increasing evidence indicates that somnambulism is associated with functional abnormalities during wakefulness and that sleep deprivation constitutes an important drive that facilitates sleepwalking in predisposed patients. Here, we studied the neural mechanisms associated with somnambulism using Single Photon Emission Computed Tomography (SPECT) with ^99m^Tc-Ethylene Cysteinate Dimer (ECD), during wakefulness and after sleep deprivation.

**Methods:**

Ten adult sleepwalkers and twelve controls with normal sleep were scanned using ^99m^Tc-ECD SPECT in morning wakefulness after a full night of sleep. Eight of the sleepwalkers and nine of the controls were also scanned during wakefulness after a night of total sleep deprivation. Between-group comparisons of regional cerebral blood flow (rCBF) were performed to characterize brain activity patterns during wakefulness in sleepwalkers.

**Results:**

During wakefulness following a night of total sleep deprivation, rCBF was decreased bilaterally in the inferior temporal gyrus in sleepwalkers compared to controls.

**Conclusions:**

Functional neural abnormalities can be observed during wakefulness in somnambulism, particularly after sleep deprivation and in the inferior temporal cortex. Sleep deprivation thus not only facilitates the occurrence of sleepwalking episodes, but also uncovers patterns of neural dysfunction that characterize sleepwalkers during wakefulness.

## Introduction

Sleepwalking, or somnambulism, is defined by complex behaviors arising primarily during slow-wave sleep (SWS), with the subject displaying altered consciousness, impaired judgment, mental confusion, and decreased responsiveness to environmental stimulation [1, 2]. These behaviors may be as simple as moving objects or walking in the room, or can involve elaborate actions such as preparing food or getting ready for work in the middle of the night. Somnambulism affects approximately 2–4% of adult individuals [3], in whom it constitutes a risk for serious injuries to oneself or other household members [4].

Despite the high prevalence and serious consequences of somnambulism, the neural mechanisms underlying this condition remain poorly understood. Sleepwalking is usually considered as a disorder of arousal, i.e. resulting from an incomplete transition from SWS to wakefulness [5]. Most studies of somnambulism have investigated either the episodes themselves or the sleep period immediately preceding episode onset [6, 7]. This contrasts with the paucity of reports focusing on daytime function abnormalities in sleepwalkers. Yet, recent data emphasize the need for studying the consequences of chronic somnambulism on wakefulness. For example, several studies demonstrated that sleepwalkers show excessive daytime somnolence, both subjectively [8, 9] and objectively [10], arguing that daytime sleepiness might constitute an intrinsic feature of the somnambulism phenotype. Beyond mere sleepiness, neurophysiological data also demonstrate functional impairment of the motor cortex during daytime in sleepwalkers [11]. However, a systematic investigation of neural activity across the whole brain during wakefulness in sleepwalkers has yet to be performed.

Daytime sleepiness, which is known to reflect increased sleep propensity, may also play a role in the appearance of subsequent sleepwalking episodes. Indeed conditions that further increase the homeostatic pressure for sleep, such as sleep deprivation, are known to raise the likelihood of somnambulistic events during the subsequent sleep period. Sleep deprivation increases both the number and the complexity of sleepwalking events during recovery sleep [12]. This suggests that sleepwalkers present an abnormal reaction to factors that promote sleep propensity, sleepiness, or that deepen sleep. The neural processes underlying the inducing role of sleep deprivation on sleepwalking are not clearly identified. In particular, no study has investigated the daytime effects of sleep deprivation on brain function in sleepwalkers.

Therefore, studies of brain activity during wakefulness and after sleep deprivation in sleepwalkers are warranted to clarify pathophysiological mechanisms underlying this sleep disorder and its daytime consequences. Functional neuroimaging has proven to be an essential and non-invasive method for the study of neural processes underlying sleep disorders in humans [13]. In particular, studies of brain perfusion during wakefulness with Single Photon Emission Computed Tomography (SPECT) have brought important contributions to the pathophysiology of sleep disorders such as narcolepsy [14] and REM-sleep behavior disorder [15], by identifying dysfunctional neural circuits during wakefulness. In the field of somnambulism, only one brain imaging study has been published and reported brain perfusion patterns associated with an episode of sleepwalking in a single individual [16]. No imaging study of brain function has been performed during wakefulness or after sleep deprivation in a group of sleepwalkers.

The goal of this study was to further investigate the neural mechanisms underlying the pathophysiology of somnambulism by studying brain perfusion patterns during wakefulness in adult sleepwalkers compared to a control group of matched good sleepers using high-resolution SPECT with ^99m^Tc-Ethylene Cysteinate Dimer (ECD). In addition, the role of sleep deprivation in somnambulism was investigated by using ECD-SPECT during wakefulness immediately following a night without sleep. We hypothesized that sleepwalkers would demonstrate altered brain perfusion during wakefulness predominantly after sleep deprivation, in line with the aggravating role of the sleep-deprived state on somnambulistic manifestations.

## Materials and Methods

### Subjects

Suspected sleepwalkers were either referred to the sleep clinic at the Sacré-Coeur Hospital by their attending physicians or recruited through local advertisements. They all underwent a clinical interview with a sleep specialist to confirm the diagnosis of idiopathic somnambulism according to standard diagnostic criteria [1]. In addition, they had a full night of polysomnographic (PSG) recording that included a full EEG montage and standard monitoring of respiration to rule out other disorders such as nocturnal epilepsy and sleep apnea syndrome (apnea-hypopnea index (AHI) > 10/h). All sleepwalkers were more than 18 years old, and had a history of chronic (> 3 years) and frequent (> 1 episode / month) somnambulism that was not of a drug-induced, traumatic or neurological origin. Exclusion criteria were psychiatric conditions according to DSM-V, other sleep and neurological disorders, as well as current use of psychotropic drugs. Eleven subjects met the criteria for inclusion and agreed to participate in the study. Twelve healthy subjects without sleep complaint, matched for age and gender, were recruited as a control group, through local advertisements. Control subjects also underwent a full night of PSG recording to exclude the presence of sleep disorders (e.g. sleep apnea syndrome), as well as one night of total sleep deprivation. In sleepwalkers as well as in controls, the full night of PSG and the night of total sleep deprivation were both performed at the sleep laboratory, and were separated by an interval of at least one week. All subjects were monitored with EEG, EOG and chin EMG during the sleep deprivation period, in order to ensure they remained awake throughout the whole protocol. Demographic and PSG parameters for both groups were compared using student *t* tests for continuous variables and chi-square tests for categorical variables. The protocol was approved by the ethics committee of the Sacré-Coeur Hospital–Université de Montréal, and all participants provided written consent.

### SPECT data acquisition and analysis

SPECT scans with ^99mc^Tc-ECD were performed in a high-resolution SPECT scanner yielding a 2 mm FWHM resolution (NeuroFocus, NeuroPhysics, Shirley, MA). Sleepwalkers and controls were scanned in two sessions during morning wakefulness, a first time after a full night of sleep (baseline), and a second one (at least a week apart) after a night of total sleep deprivation (SD). In each session, a freshly prepared unidose of 750 MBq of ^99mc^Tc-ECD was administered followed by a saline flush of 30cc while the subject was lying awake in the preparation room next to the scanner. Approximately 15 min later, subjects were scanned in the NeuroFocus SPECT scanner with a static 20-min acquisition according to the manufacturer’s prescribed procedure. Throughout the scanning procedure, subjects were instructed to relax and remain awake with their eyes open. After standard reconstruction (filtered back projection, subtraction of 50% of the Compton window from the peak window) and attenuation correction (noniterative Chang algorithm), SPECT data were processed using Statistical Parametric Mapping 8 implemented in Matlab (version 7.11). All studies were registered and normalized to a standard SPECT template, and smoothed with a 14-mm FWHM filter. Global normalization was performed using proportional scaling. Grey matter threshold was set at 1.0 (instead of the 0.8 default SPM value), in order to minimize the risk that findings would be an artefact of global mean normalization [17].

Differences in regional cerebral blood flow (rCBF) between groups (sleepwalkers vs controls) at baseline were assessed using a two-sample t-test. Then, in order to evaluate the effects of sleep deprivation, a repeated measures ANOVA (flexible factorial design) was performed with group as the between-subject factor and session as a within-subject factor. T-contrasts were generated to evaluate the main effect of group (i.e., comparison between sleepwalkers and controls including both baseline and SD sessions), and to decompose the group by session interaction (effect of sleep deprivation on the group comparison). To evaluate whether brain perfusion patterns in sleepwalkers during wakefulness could be related to sleep fragmentation during the preceding night, correlations between rCBF and PSG indices of fragmentation (arousal index, number of sleep stages shifts from SWS to wakefulness) were assessed in sleepwalkers at baseline. To evaluate whether brain perfusion patterns in sleepwalkers during wakefulness could be related to daytime sleepiness levels or sleepwalking intensity as reflected by the frequency of episodes, correlations between rCBF and Epworth sleepiness score, as well as between rCBF and episode frequency, were assessed in sleepwalkers at baseline and after sleep deprivation. For all contrasts, significance was set at p < 0.05 after correction for multiple comparisons on small volumes (sphere, 10mm). Significant results were overlaid on a MRI template for localization of rCBF changes.

## Results

Demographic and PSG parameters for both groups are shown in Table 1. Out of the 11 sleepwalkers studied, 10 were scanned during baseline wakefulness (1 was lost due to technical problems) and 8 were scanned after SD (3 were not acquired because of technical issues or conflicts with participants’ schedule). Twelve controls were scanned during baseline and 9 of them were also scanned after SD (3 were lost because of technical issues or conflicts with participants’ schedule).

**Table 1:** Demographic and sleep characteristics.

	Sleepwalkers (n = 11)	Controls (n = 12)	P value
Male	8 (72.7%)	9 (75%)	1
Age (years)	28.9 (5.6)	29.4 (4.4)	0.8
Education (years)	16 (1.1)	16.5 (2.1)	0.5
ESS^†^	8.7 (5.9)	N/A	N/A
Episode frequency^§^	2-3/week (1/month-1/night)	N/A	N/A
Sleep latency (min)	12.5 (9.5)	18.4 (8.5)	0.1
Total sleep time (min)	406.2 (43.6)	395.1 (33.2)	0.5
Sleep efficiency (%)	87.6 (8.4)	87.2 (6.9)	0.9
Stage N1 (%)	10.6 (4.7)	11.3 (4.4)	0.7
Stage N2 (%)	55.9 (5.5)	57.5 (7.7)	0.6
Stage N3 (%)	15.5 (6.9)	15.3 (6.2)	0.9
Stage REM (%)	18 (3.3)	15.9 (3.1)	0.1
Arousal index (events/hour)	5.8 (2.4)	6.3 (3.2)	0.7
Number of N3-wake transitions^#^	2.7 (1.3)	1.3 (1.6)	0.04
AHI^##^ (events/hour)	1.5 (2.7)	1.4 (1.7)	0.9
PLMSi^###^ (events/hour)	10.4 (7.5)	4.2 (5.1)	0.03

^†^Epworth Sleepiness Scale; values missing for 2 subjects

^§^median episode frequency (range); values missing for 2 subjects

^#^number of sleep stage shifts from N3 to wakefulness

^##^apnea-hypopnea index

^###^index of periodic limb movements during sleep

**Fig 1:**
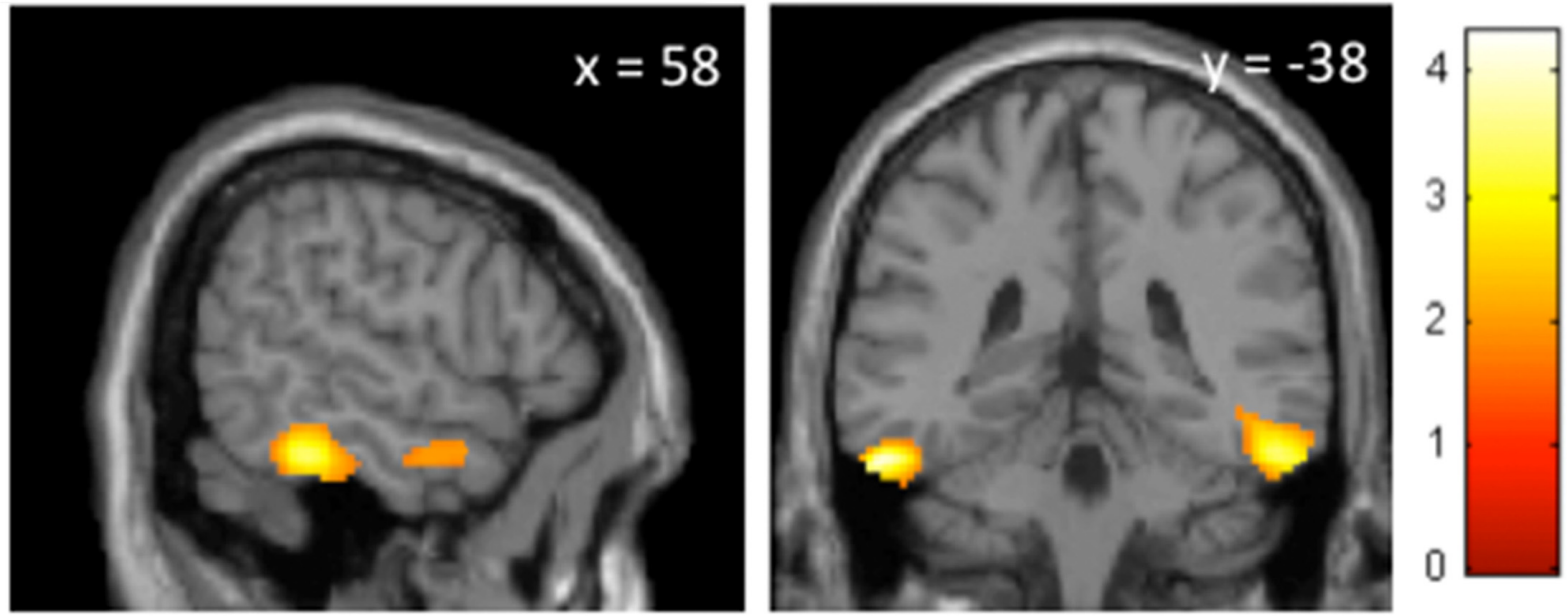
Effects of sleep deprivation on brain perfusion patterns during wakefulness in sleepwalkers compared to controls. Brain perfusion decreases after the sleep deprivation session, in sleepwalkers compared to controls, were located in the inferior temporal gyrus, bilaterally (x = -48mm y = -22mm z = -26mm, p = 0.05, t = 2.69; x = 58mm y = -38mm z = -24mm, p = 0.04, t = 2.80), as displayed on sagittal (left panel) and coronal (right panel) sections. The level of section is indicated on the top of each panel (*x* and *y* coordinates, in mm). The color scale indicates the range of *t* values for this contrast. Results were significant at p < 0.05, after correction for multiple comparisons on 10-mm spherical volumes centered on published coordinates [15].

Group comparison of SPECT data showed no difference of brain perfusion between sleepwalkers and controls at baseline. Group effects from pooled baseline and SD conditions revealed significant hypoperfusion during wakefulness in sleepwalkers compared to controls in the inferior temporal gyrus bilaterally (x = 58mm y = -38mm z = -26mm, p = 0.015, t = 3.20; x = -56mm y = -30mm z = -26mm, p = 0.024, t = 2.99) and right hippocampus (x = 28mm y = -20mm z = -6mm, p = 0.030, t = 2.89). No area showed increased perfusion in sleepwalkers. Interactions between group and session showed that, for the SD condition, decreased perfusion was only observed in the inferior temporal gyrus bilaterally in sleepwalkers compared to controls (x = -48mm y = -22mm z = -26mm, p = 0.05, t = 2.69; x = 58mm y = -38mm z = -24mm, p = 0.04, t = 2.80) (Fig 1). Finally, there was no significant correlation between rCBF during baseline wakefulness and PSG indices of sleep fragmentation (arousal index or number of sleep stage shifts from SWS to wakefulness) during the preceding night in sleepwalkers. Likewise, there was no significant correlation between rCBF and Epworth sleepiness scores, or between rCBF and episode frequency, in sleepwalkers during baseline wakefulness or after sleep deprivation.

## Discussion

Using functional neuroimaging with SPECT during wakefulness, we found decreased regional brain perfusion in adult sleepwalkers compared to controls in the inferior temporal gyrus after sleep deprivation. These results constitute the first neuroimaging evidence of altered brain function in somnambulism during wakefulness, and the first imaging study conducted in a group of sleepwalkers. In addition, the present results highlight the role of sleep deprivation in the expression of somnambulism: sleep deprivation not only facilitates the occurrence of sleepwalking episodes in predisposed patients but also reveals altered functional brain patterns during sleepwalkers’ wakefulness.

In their ^99m^Tc-ECD SPECT study conducted on a 16-year-old man, Bassetti and colleagues examined brain perfusion during an episode of sleepwalking and during SWS outside the episode [16]. They observed that the sleepwalking episode compared to baseline SWS was associated with a higher perfusion in the anterior cerebellum and posterior cingulate cortex, a pattern that is reminiscent of wakefulness [18]. In contrast, when comparing the episode in this sleepwalker to a wakefulness state in control good sleepers, they showed a lower perfusion of frontoparietal association areas during the episode, a pattern that resembles normal SWS [18]. Hence the presence of both decreases and increases of rCBF during a somnambulistic episode was interpreted as reflecting a dissociated state in which functional signs of both SWS and arousal states coexist [16].

The variability in the clinical presentation of sleepwalking events from an individual to another might constitute a challenge in the study of consistent brain activation patterns during the actual somnambulistic episodes across subjects. This emphasizes the importance of investigating brain function outside the behavioral manifestations, and particularly during wakefulness, as previously performed for other sleep disorders [14, 15]. Here we demonstrate functional brain abnormalities that are consistent across a group of sleepwalkers compared to controls. Importantly, our results indicate that beyond the time window of actual sleepwalking events, somnambulism is characterized by altered perfusion in segregated brain areas. The fact that functional changes were observed during wakefulness is in agreement with recent findings emphasizing the daytime correlates and consequences of somnambulism. In addition, the absence of significant correlations between these brain perfusion patterns and indices of sleep fragmentation during the previous night supports the presence of intrinsic daytime neural dysfunctions independently of preceding sleep disruption. Furthermore, their absence of correlation with episode frequency indicates that these changes were not explained either by this index of sleepwalking intensity.

A first line of evidence for daytime dysfunction arises from studies evaluating the level of sleepiness in sleepwalkers. Subjective daytime somnolence, as assessed by the Epworth sleepiness scale, was found to reach pathological levels in almost half of sleepwalkers investigated [8, 9]. This sleepiness score was however not associated with the observed brain perfusion changes in the present study. Daytime mean sleep latency during naps was also significantly lower in patients with somnambulism, even after nights without episodes, indicating objectively high sleepiness levels in this population [10]. Data supporting the existence of daytime functional anomalies in sleepwalkers have also been obtained in a study of cortical excitability. Oliviero and colleagues used transcranial magnetic stimulation (TMS) to evaluate the excitability of the motor cortex during wakefulness in a group of 8 sleepwalkers and found a significant reduction of several TMS parameters reflecting the activity of inhibitory circuits [11]. Their findings suggest that somnambulism is characterized by an impairment of the inhibitory control of motor systems, which is detectable during wakefulness. Furthermore, recent evidence suggests that sleep deprivation increases sleepwalkers’ behavioral impulsivity during wakefulness differentially than good sleepers in a continuous performance task paradigm [19].

In our study, we observed consistent functional abnormalities not in the motor cortex but in posterior association cortices, namely the inferior temporal cortex bilaterally. While the decreased perfusion in the hippocampus was not confirmed when inspecting the baseline and sleep-deprived conditions separately, hypoperfusion of the inferior temporal cortex was observed under the grouped conditions and after sleep deprivation. Interestingly, no significant difference was found between controls and sleepwalkers during baseline wakefulness after a complete night of sleep. The specificity of altered brain perfusion patterns to the sleep-deprived state emphasizes the importance of increased homeostatic pressure on the expression of the somnambulistic phenotype. It is well established that sleep deprivation creates a vulnerability window for the manifestation of sleepwalking episodes in predisposed individuals: the frequency of episodes is increased during recovery sleep following sleep deprivation, and the complexity of the episodes is also enhanced [12]. Here, we show that sleep deprivation also allows for the detection of patterns of neural dysfunction characterizing the waking state of adults suffering from chronic somnambulism. The exact contribution of the inferior temporal cortex to the pathophysiology of sleepwalking remains unclear. This region is involved in the cognitive processing of visual information such as the identification and recognition of objects [20]. Interestingly, previous functional neuroimaging studies have shown that this region is particularly vulnerable to sleep deprivation: activity in this region was found decreased in the sleep-deprived state proportionally to performance decrease at a non-verbal recognition task [21]. Our results might thus reflect the preferential vulnerability of the inferior temporal cortex to sleep deprivation in somnambulism. The extent to which this decreased perfusion in wakefulness impacts behavior remains to be established, given the lack of data systematically investigating cognitive performances and visual processing in sleepwalkers. It is finally important to remind that sleepwalkers frequently present visual hallucinatory perceptions during their episodes [22]. Those perceptions might consist for instance of threatening visual content (e.g. presence of an intruder or animals threatening to harm them), which triggers the specific behavior observed during the sleepwalking episode (e.g., trying to escape the immediate danger). Thus, it would be interesting to relate our findings of altered perfusion in visual association areas after sleep deprivation with the visual phenomenology accompanying sleepwalking episodes. This could be achieved through a systematic study of visual mental content in the sleepwalking episodes during baseline nights as compared to recovery nights after sleep deprivation, with the aim of evaluating whether sleep deprivation increases the presence and intensity of hallucinatory perceptions accompanying such episodes.

## Conclusion

This first neuroimaging study performed on a group of sleepwalkers highlights the existence of functional brain changes during wakefulness in chronic somnambulism. These changes consisted in brain perfusion decreases in sleepwalkers compared to controls, following sleep deprivation, and localized in the inferior temporal cortex. Whether such functional abnormalities during daytime constitute a prelude to the development of somnambulistic manifestations during sleep or rather reflect the daytime consequences of chronic sleep disruption in sleepwalkers remain to be further investigated. Given the relatively limited sample size of the present study, future neuroimaging studies should replicate and confirm these findings in larger samples of chronic sleepwalkers. It will also be interesting to combine functional neuroimaging studies of somnambulism with cognitive and behavioral assessments to further characterize the potential impact of these abnormal brain responses on daytime function in chronic sleepwalkers. Finally, despite the challenges associated with functional brain imaging acquisitions during sleep, studies of somnambulism could benefit from further functional neuroimaging recordings during SWS in order to explore the conceptualization of somnambulism as a disorder of SWS [6, 7].
